# Does outsourcing enable the survival of good care homes? A longitudinal analysis of all care homes in England, 2011–2023

**DOI:** 10.1136/bmjph-2024-001227

**Published:** 2024-07-29

**Authors:** Anders Malthe Bach-Mortensen, Benjamin Goodair, Michelle Degli Esposti

**Affiliations:** 1 Department of Social Policy and Intervention, University of Oxford, Oxford, UK; 2 Roskilde University, Roskilde, Denmark; 3 Department of Social Sciences and Business, University of Michigan, Ann Arbor, Michigan, USA

**Keywords:** Social Medicine, Public Health, Community Health

## Abstract

**Background:**

It is unclear whether outsourcing has enabled the growth and survival of the best care homes, as intended. We aimed to test whether ownership (for-profit, public and third sector (non-profit)) influences determinants of closure among all care homes in England, 2011–2023.

**Methods:**

We created a dataset of all care homes from 2011 to 2023 (29 548 care homes and 8346 closures) and Care Quality Commission inspections from 2014 to 2023 (n=65 726). Using logistic regression, we investigated determinants of care home closures including care home characteristics (eg, number of beds), service registrations (eg, dementia and nursing), quality (inspection ratings) and area deprivation. We then tested for interaction effects; specifically exploring (1) whether the determinants of closure vary by ownership and (2) whether quality differences between active and closed for-profit and third sector/public homes vary by area deprivation.

**Results:**

The prevalence of for-profit care homes increased from 2011 to 2023. Ownership was a key determinant of care home closure; public and third sector care homes were almost twice as likely to close than for-profit providers (OR 1.8; 95% CI 1.44 to 2.24, p<0.001 and OR 1.6; 95% CI 1.37 to 1.76, p<0.001, respectively). Although care quality was also a significant determinant of closure, this association varied by care home ownership. For example, public and third sector homes rated ‘good’ were 7.6 percentage points (p<0.001) and 5.9 percentage points (p<0.001) more likely to close than for-profit homes with the same rating. Lastly, the quality of for-profit homes is heavily influenced by area deprivation, and the best active homes in deprived areas are operated by public and third sector providers.

**Conclusion:**

Our findings suggest that outsourcing of care services has not promoted the survival of the best care homes and may have adverse effects on the equity and accessibility of care. This calls for a reassessment of current policies to ensure that vulnerable populations have continued access to adequate quality of care.

**WHAT IS ALREADY KNOWN ON THIS TOPIC**

- Most adult social care services in England are outsourced to the private (for-profit and third sector (non-profit)) organisations. The impact of this development in terms of whether outsourcing promotes the growth and survival of the best care homes is unclear.

**WHAT THIS STUDY ADDS**

- By analysing all care home closures 2011–2023, this study shows that care homes were more likely to close if they are (a) operated by the public or third sector, (b) located in a deprived area or (c) rated poorly by regulator inspections. Our analysis further shows that public and third sector homes rated the same as for-profit homes are generally more likely to close.

**HOW THIS STUDY MIGHT AFFECT RESEARCH, PRACTICE OR POLICY**

- The quality of for-profit homes is heavily influenced by area deprivation, and the best homes in deprived areas are operated by public and third sector providers. This suggests that the outsourcing of care services has not promoted the survival of the best non-profit care homes and may have adverse effects on the equity and accessibility of care.

## Introduction

 The ageing population has resulted in increased demand for care, which has made the organisation, regulation and funding of adult social care a policy priority.^1^ Many countries are struggling to finance this demand and are increasingly turning to the market in an attempt to optimise care quality and increase the capacity of the sector. This is typically achieved by outsourcing services to a competing market of public and private (both for-profit and non-profit third sector) social care providers.^2^ However, research suggests that decades of outsourcing have not delivered in terms of improved quality of care or capacity.^3 4^ Understanding why markets are currently not delivering their intended benefits is crucial for the future provision of social care.

Outsourcing not only functions through a competitive market, but it also shifts provision from public to privately owned (often for-profit) providers. In England, more than 95% of all care home provision is private, of which more than 85% is run by for-profit services.^5^ Private sector provision of adult social is also on the rise internationally.^6 7^ Most care homes (60%) in England serve a mix of state-funded and self-funded residents, and there are very few homes (less than 1.5%) that only accommodate residents funding their own care.^8^ This means that most care homes receive income from both private self-payers and the state. However, the population of self-funding residents is increasing, and, in 2023, this group constituted 37% of all care home residents.^8^ The impact of this client mix in terms of the supply and quality of provision is not well understood, in part because of a scarcity of care home level data on the funding status of care home residents.

Research on the impact of for-profit ownership has found that for-profit care homes deliver worse quality than public and third sector (non-profit) care homes.^39^,^11^, and the evidence suggests that the pursuit of profit influences the behaviour of providers in ways that negatively impact the quality of care and residents.^12^ However, it is unclear whether the profit motive also impacts the performance of competition, regulation and market oversight on a broader scale.

A key reason for this knowledge gap is that little attention has been given to the patterns of care home closures among for-profit, public and third sector providers. In England and internationally, outsourcing was intended to improve care supply and quality and to reduce costs through competition and by promoting consumer choice among residents.^12 13^ If these intentions were realised, one would expect outsourcing to result in the closure of poorly performing providers, and the survival of the best quality services. While existing research shows that ownership matters in terms of incentives and performance,^4 14 15^ little is known about whether outsourcing has enabled ‘good’ providers to thrive and poor-performing care homes to close, as would be expected in a well-functioning market.

Care home closures play a crucial role in the functioning of the sector, serving as a mechanism to regulate, adjust to changing resident preferences and improve overall performance. Closures are a multifaceted outcome. It can represent a reactive mechanism that removes poorly performing operators to protect residents from poor services while closures also have the potential to cause disruption and trauma to residents and create substantial added costs to local authorities (LAs).^16 17^ It can also serve as a way for the sector to adapt to shifting preferences among care residents, such as domiciliary care. The closure of underperforming care homes is crucial for protecting residents, but if the sector is poorly regulated, closures may be driven by factors unrelated to care quality in ways that can harm accessibility and resident equity. For instance, if the financial survival of care homes is not determined by service quality, but by being able to attract affluent residents, the accessibility of care provision will depend on the prosperity of the area rather than residents’ needs.

Research on the US care homes has found evidence to support this concern. Several large-scale observational studies have found that area poverty and proportion of Medicaid residents impact the risk of closure.^18 19^ Further, there is evidence suggesting that the quality of services is worse in deprived areas.^20^ US research has also found that for-profit care homes are less likely to close than public and third sector homes^21^. Yet, the relationship between ownership and area deprivation is not well understood. For example, it is unclear whether for-profit homes are less likely to close simply because they most frequently operate in less deprived areas. In such cases, care home closures may reflect area deprivation rather than care home quality.

While equivalent research in the UK context is limited, the current evidence suggests that home closures in England are influenced by both care home quality and area characteristics.^22 23^ Using care home data in England from 2008 to 2010, Allan and Forder found that higher inspection ratings and lower levels of local competition were both linked to a lower risk of closure.^23^ A more recent analysis looking at the association between domiciliary care supply and closures using 2014–2016 data, also found that higher quality, as measured by Care Quality Commission (CQC) inspections, was associated with a lower risk of closure.^22^ Further, the analysis did not establish an association between increases in domiciliary care and care home closures, which suggests that at-home services have not substituted residential care. Both studies identify a link between inspection ratings and risk of closure, but the investigated period of each study is relatively short (2 years) and is, therefore, unable to reveal patterns and changes over time.

None of the existing work analyses how determinants of closure vary according to ownership. This is an important research gap, as the provision landscape in England has undergone a gradual ownership transformation in favour of the for-profit sector. It is not clear if this development is due to a disproportionate number of closures among public and third sector homes, higher rates of for-profit openings, nor whether the determinants of closure vary by ownership. Industry regulators have continuously raised concerns that care access is worsening in the most deprived parts of the country.^24^ This trend has not been evaluated against the growth in outsourcing in adult social care.

### Research objectives

For-profit care home provision has increased over time, but there is limited evidence on whether outsourcing has enabled the survival of the best providers, and if this depends on the area of operation. The aims of this article are to (1) identify how inspection ratings, area deprivation and ownership are associated with care home closure,(2) to test if these effects vary by ownership and (3) explore whether quality differences between active and closed for-profit and third sector/public homes vary by area deprivation.

## Materials and methods

### Data

We created a novel dataset tracking all care home closures and ownership changes, 2011–2023, and CQC inspection reports, 2014–2023. Data on CQC inspection ratings are only available from 2014 due to the change in inspection framework that year.^25^ We retrieved this information from the CQC Application Programming Interface (API),^26^ which stores detailed data on all active and closed care homes, including number of registered beds, start and closure date, regulated activities and postal codes.

#### Care home closure

We used the location ID and closure date to distinguish between different types of care home closures. A care home location ID will change if (1) the location closes, (2) the location is taken over by a different provider or (3) the location changes address. To separate care home closures from provider takeovers, legal entity changes and address changes, we created a unique identifier that tracks the life course of each care home. Our main outcome in this paper is complete care home closure - that is, when a care home ceases to operate.

#### Inspection results

We retrieved and coded all inspection results from 2014 to 2023. We used the CQC API to download all inspection results over time for each care home.^26^ The CQC evaluates care homes based on their five standards: ‘safety’, ‘effectiveness’, ‘caring’, ‘responsiveness’ and if a service is ‘well-led’, which are summarised by an overall rating. All ratings are on a 4-point scale: ‘inadequate’, ‘requires improvement’, ‘good’ and ‘outstanding’. The results presented in the manuscript focus on the overall assessments, but the results are similar across all inspection domains (see online supplemental figure A3).

#### Ownership

We coded care home ownership by categorising all registered charities and charitable companies as ‘third sector’ (non-profit), and all private companies, partnerships and individual providers without a charity number as ‘for-profit’. All council, National Health Service (NHS) and municipality care homes were coded as ‘public’. To account for variation within the for-profit category, we separately control care homes run by individuals and partnerships versus private companies. This means that our results primarily relates to for-profit companies rather than homes operated by individuals or in partnerships.

#### Area deprivation

We used the postcode to connect each care home location to a lower layer super output area (LSOA) and linked this to the English indices of deprivation. We use the Income Deprivation Affecting Older People Index 2015 and 2019 scores (depending on the opening, closure and inspection dates),^27^ which reflects the proportion of people above 60 experiencing income deprivation living in each LSOA.

The final dataset included a total of 65 726 inspection reports from 2014 to 2023. We included 29 548 unique location IDs, which translates into 23 022 unique care homes when accounting for provider takeovers and address changes.

### Patient involvement

Patients were not involved in the design, conduct, reporting or dissemination plans of this research.

### Statistical analysis

First, we analysed key determinants of care home closure from 2011 to 2023. We used logistic regression with fixed effects for LA and year of inspection to model the effects of ownership (for-profit, public and third sector), care quality (‘inadequate’, ‘requires improvement’, ‘good’ and ‘outstanding’) and area deprivation^27^ on care home closures (n=8346). In each model, we control for care home and area characteristics. These include years of registration, number of beds, if the care home includes nursing, and what type of residents a care home is registered to accommodate (eg, older people, dementia and disabled residents). All standard errors (SEs) are clustered at the unique identifier we constructed, which takes care home takeovers over time into account, and at the unique provider ID to account for multifacility care home chains.

Second, we tested if the effects of area deprivation and inspection rating on care home closure varied by ownership status by including additional interaction terms in the above logistic regression models. To allow for non-linear interaction effects,^28^ the logistic regression results are presented as marginal effects and predicted probabilities, which are less sensitive to model specification than odds ratio (ORs).^29 30^ We further calculate the differences in predicted probabilities for each interaction model to test if these are statistically significant across ownership, inspection ratings and area deprivation.

Third, we ran a three-way interaction of closure status (closed/active), area deprivation and ownership using logistic regressions^31^ with CQC inspection ratings as the outcome. This allowed effects to vary across all three variables and for us to test for non-linear associations.^28^ It thus enabled us to test how the impact of ownership and area deprivation on quality varies by whether a home is active or closed. To ease the interpretation of these results, we derived a binary variable for inspection ratings (good vs poor quality), collapsing ‘good’ and ‘outstanding’, and ‘inadequate and ‘requires improvement’, respectively. The results are similar when analysing the original ratings in ordinal logistic regression models (see online supplemental figure A6 and A7).

This model is visualised as average predicted probabilities^32^ in figure 3, and the full logistic regression output is in online supplemental table A4 . The model controls for the full range of covariates and fixed effects as described above.

### Sensitivity analysis

We reproduce all our analyses with the individual care homes as the unit of analysis (see online supplemental figure A3 and table A1). We also conduct all of our interaction effects with deprivation percent as a continuous variable (rather than categorical), to test whether the slopes varying by ownership are statistically significant (see online supplemental table A3). Last, we run multiple variations on our main regression results to test whether our main coefficients vary according to quality outcome and model specification (see online supplemental figure A6 and A7).

## Results

### Descriptive results

As of October 2023, 85.41% (12619/14774) of all active care homes in England were operated by for-profit sector (table 1). More third and public sector homes closed relative to for-profit homes. Of the closed care homes with an inspection rating, 20.46% and 24.98% of the closed homes were rated ‘inadequate’ or ‘requires improvement’. Most (77.31%) of the currently active care homes are rated ‘good’, with around 18.5% being rated ‘inadequate’ or ‘requires improvement’. Areas with higher deprivation had a greater proportion of closed care homes.

**Table 1:** Characteristics of active and closed homes as of October 2023

	Closed care homes, 2011–2023				Active care homes, October 2023			
	All(n=8346)	For-profit(73.47%)	Local authority (8%)	Third sector(18.52%)	All(n=14774)	For-profit(85.41%)	Local authority (2.72%)	Third sector(11.87%)
Organisation type								
Individual/partnership	20.42%(1712/8346)	27.92%(1712/6132)	0	0	7.19%(1062/14774)	8.42%(1062/12619)	0	0
Registration characteristics								
Mental health needs	22.42%(1870/8346)	24.46%(1500/6132)	19.46%(130/668)	15.78%(244/1546)	27.57%(4077/14774)	28.91%(3648/12 619)	24.38%(98/402)	18.88%(331/1753)
Disabled	26.52%(2212/8346)	26.4%(1619/6132)	33.68%(225/668)	23.93%(370/1546)	39.79%(5884/14774)	39.87%(5031/12 619)	51.74%(208/402)	36.79%(645/1753)
Dementia	33.92%(2829/8346)	38.44%(2357/6132)	36.23%(242/668)	15.2%(235/1546)	52.01%(7690/14774)	55.06%(6948/12619)	54.48%(219/402)	29.83%(523/1753)
Care home characteristics								
Includes nursing (%)	17.98%(1499/8346)	21.3%(1306/6132)	5.84%(39/668)	9.96%(154/1546)	27.67%(4088/14774)	29.38%(3707/12 619)	12.69%(51/402)	18.82%(330/1753)
Months of registration (SD)	56.86(40.90)	57.52(40.38)	42.35(35.98)	60.30(43.58)	115.87(50.17)	112.71(50.94)	126.47(48.56)	136.23(38.45)
Care home beds (SD)	19.48(20.23)	21.14(21.56)	18.63(14.53)	13.25(14.98)	30.8286(25.31)	32.02(25.55)	25.10(20.18)	23.54(23.15)
Latest overall rating								
Inadequate	20.46%(820/4007)	24.86%(778/3129)	2.33%(4/172)	5.38%(38/706)	1.24%(178/14309)	1.36%(166/12195)	.52%(2/402)	.58%(10/1729)
Requires improvement	24.98%(1002/4007)	26.72%(836/3129)	20.35%(35/172)	18.56%(131/706)	17.18%(2459/14309)	18.2%(2219/12195)	10.39%(40/402)	11.57%(200/1729)
Good	54.09%(2168/4007)	48.13%(1506/3129)	75.58%(130/172)	75.21%(531/706)	77.31%(11062/14309)	76.14%(9285/12195)	85.71%(330/402)	83.69%(1447/1729)
Outstanding	0.35%(14/4007)	.16%(5/3129)	1.74%(3/172)	.85%(6/706)	4.26%(609/14309)	4.3%(524/12195)	3.38%(13/402)	4.16%(72/1729)
Missing inspection data*	51.95%(4333/8346)	48.97%(3003/6132)	74.25%(496/668)	54.33%(840/1546)	3.28%(486/14795)	3.36%(424/12619)	4.23%(17/402)	1.37%(24/1753)
Number of inspection reports	10092	8224(81.49%)	322(3.19%)	1546(15.32%)	47854	41441(86.60%)	1121 (2.34%)	5292(11.06%)
Area deprivation								
IDAOPI score (SD)†	18.43%(11.74)	18.41%(11.73)	20.17%(12.01)	17.69%(11.6)	15.59%(10.51)	17.7%(11)	18.71%(10.63)	14.91%(10.09)

* Because of the change in inspection framework in 2014, 52% of the closed homes do not have an inspection rating - —either because they were closed prior to receiving a rating from the 2014 framework, or if they were closed before receiving a rating.**For closed providers, we used the IDAOPI 2015 score for location closed from and the IDAOPI 2019 score for . For active providers, we used IDAOPI 2019.

† For closed providers, we used the IDAOPI 2015 score for location closed from 2011–2017 and the IDAOPI 2019 score for 2018–2023. For active providers, we used IDAOPI 2019.

IDAOPIIncome Deprivation Affecting Older People Index

**Figure 1:**
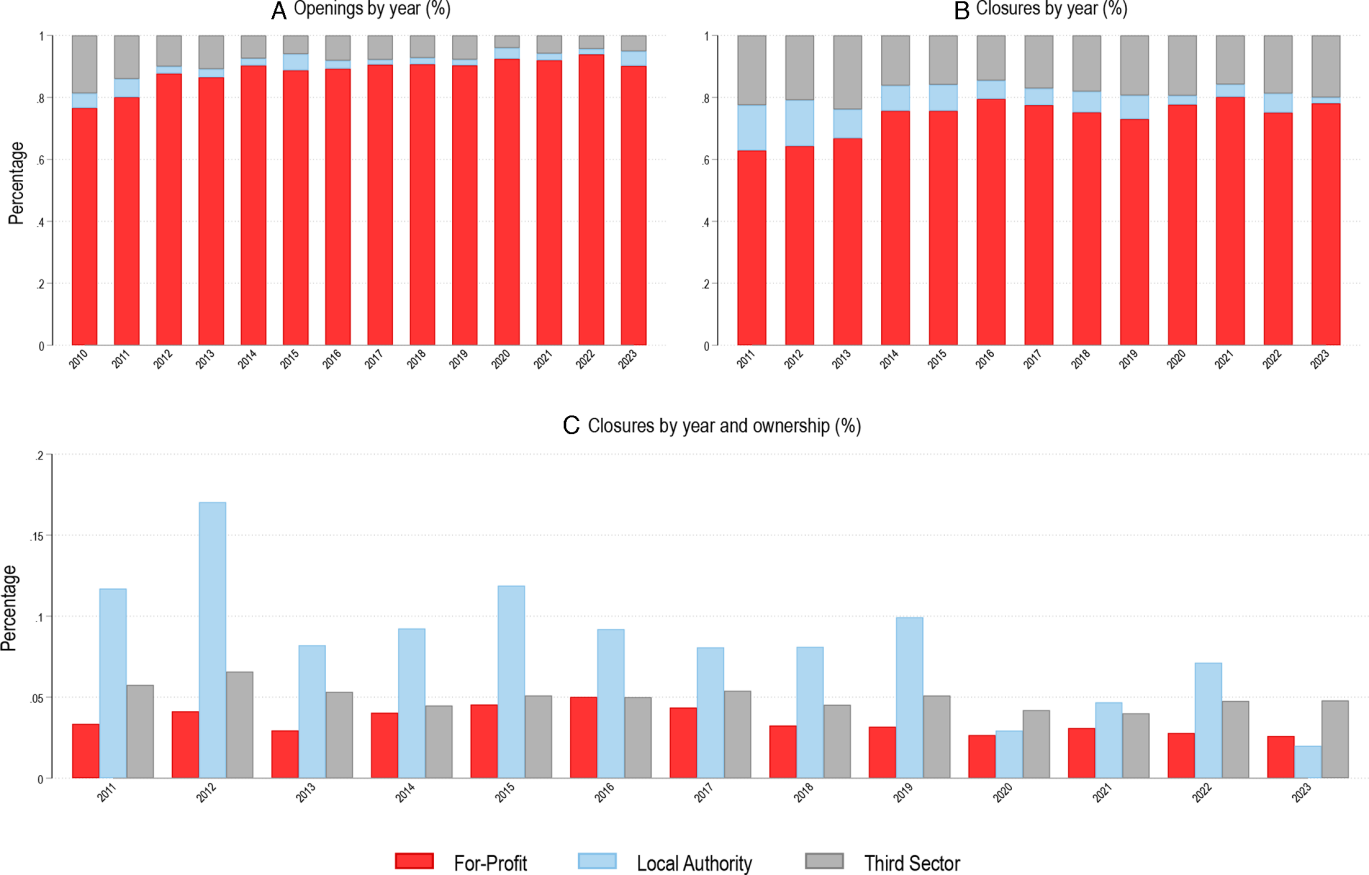
Number and proportion of care home openings and closures, 2010–2023.

From 2011 to 2023, there were 8346 care home closures (see online supplemental figure A1 for the count of closures by year). The proportion of closures relative to openings varied by ownership status (figure 1). The proportion of for-profit care home openings has increased over time and, by 2022, 93.9% of all new care homes in 2022 were for-profit, compared with 76.6% in 2010 (figure 1A). The proportion of closures has shifted over time, with for-profit homes accounting for a slightly larger proportion of all care home closures in recent years (figure 1B).

Figure 1C shows that this is because the absolute number of for-profit homes has increased, and that the proportion of yearly closures relative to the number of active homes is consistently lower among for-profit homes, even in recent years. For example, 17% and 6.6% of public and third sector homes that were active in 2012 closed that year. Therefore, from 2010 to 2023, there has been a for-profit growth with more for-profit openings and fewer closures, compared with the public and third sector. The number of active providers across ownership and the raw number of closures and openings over time can be found in online supplemental figure A1 and A2.

### Determinants of closure

Third and public sector homes were more likely to close compared with for-profit homes (p<0.001), even after controlling for quality, area deprivation, and other covariates (table 2). Ownership status was associated with the risk of closure, with public and third sector homes being 1.80 and 1.55 times more likely to be closed than for-profit care homes. Better inspection ratings were strongly correlated (p<0.001) with a lower risk of closure. Care homes rated Outstanding and Good were much less likely to have closed than homes rated with at least one Inadequate rating. Area deprivation was also associated (p<0.01) with closure, with care homes in less deprived areas less likely to close, after controlling for covariates. The results were robust to model specifications (see online supplemental table A2 and figure A3).

**Table 2:** Logistic regression results on the determinants of care home closure

	Unadjusted ORs (95% CIs)	Adjusted ORs (95% CIs)
Ownership(reference: for-profit)		
Local authority (LA)	1.427^***^	1.796^***^
	(1.156 to1.762)	(1.439 to 2.242)
Third sector	1.374^***^	1.553^***^
	(1.227 to 1.538)	(1.373 to 1.756)
Area deprivation (IDAOPI (%))	1.007^**^	1.008^**^
	(1.002 to 1.011)	(1.003 to 1.013)
Overall quality (reference: inadequate)		
Requires improvement	0.305^***^	0.281^***^
	(0.280 to 0.332)	(0.258 to 0.306)
Good	0.183^***^	0.133^***^
	(0.166 to 0.201)	(0.120 to 0.146)
Outstanding	0.0421^***^	0.0345^***^
	(0.0274 to 0.0647)	(0.0225 to 0.0530)
Observations	58 980	58 967
Pseudo R^2^	0.086	0.128
Care home clusters	18 323	18 309
LA fixed effects	Yes	Yes
Inspection year fixed effects	Yes	Yes

95% confidence intervalCIs in parentheses. *** p, ** p, * p.All standard errors are clustered at provider and care home level. .

***p<.001, **p<.01, *p<.05.

All SEs are clustered at provider and care home level.

The months of registration take provider takeovers into account and are based on the difference between the start and inspection date. The adjusted models control for registered nursing services, registered dementia services, individual/partnership organisation, registered mental health service, registered disability services, number of registered beds, care homes for older people and months of registration. See online supplemental table A1 in the appendix for full regression results.

**Figure 2:**
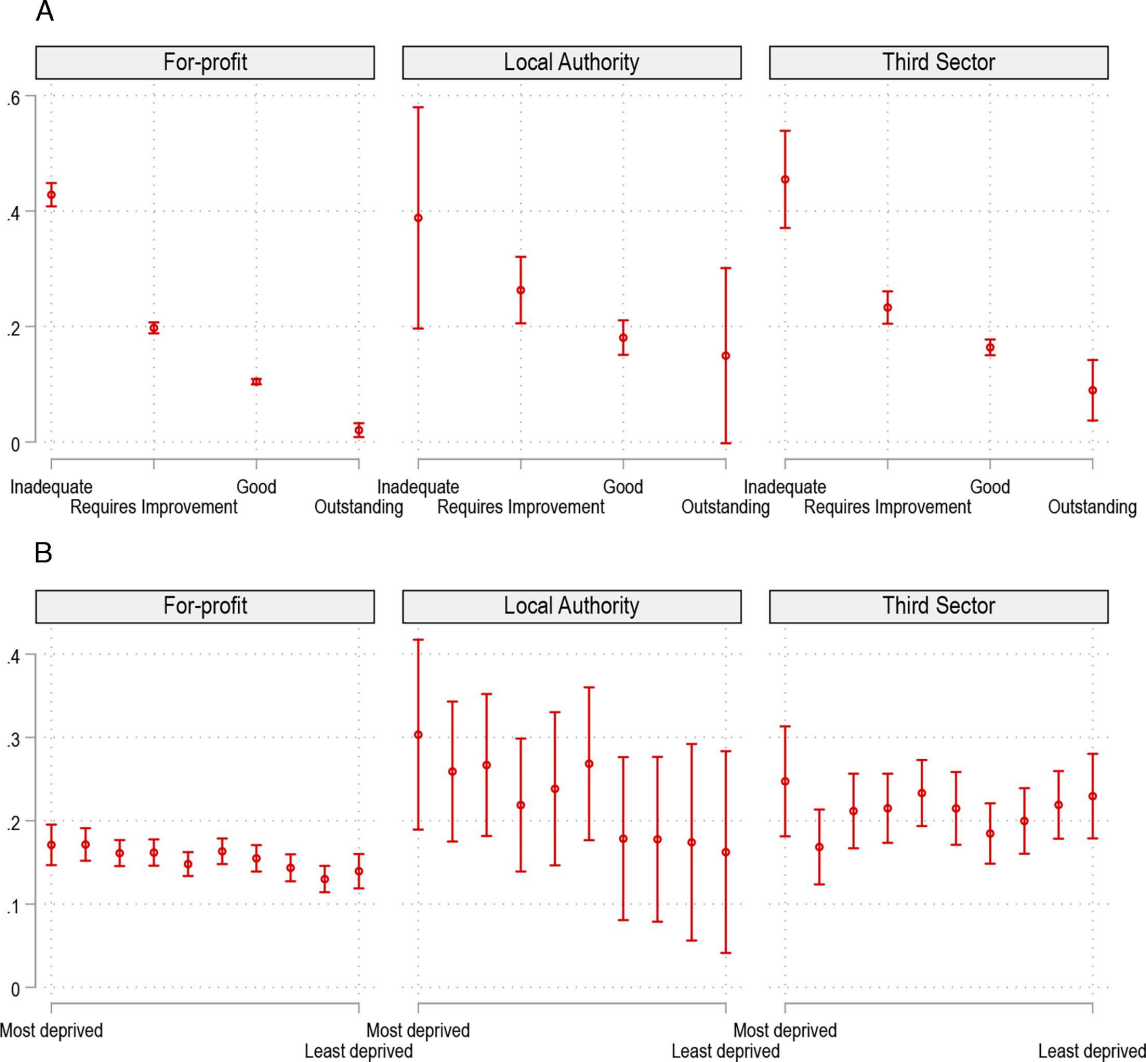
Predicted probability of closure by inspection ratings and deprivation deciles by ownership. This figure models the two-way interaction models of the risk of closure by ownership and quality (panel (A)), and ownership and area deprivation (panel (B)). The model controls for the full range of covariates and fixed effects as the adjusted model in table 2.

While ownership status, care quality and area deprivation all uniquely predicted risk of care home closure, we found that the magnitude of the effects of care quality and area deprivation varied by ownership status (figures2  3). For all ownership types, poor care quality and higher area deprivation were associated with an increased likelihood of closure (figure 2). However, the effects of care quality and area deprivation were the largest and most consistent among for-profit care homes. The predicted probability of closure was higher for public and third sector homes across all inspection ratings, except those rated ‘inadequate’ (figure 1A). The difference in closure risk was largest for homes rated ‘good’—public and third sector homes rated ‘good’ were 7.6%-points (p<0.001) and 5.9%-points (p<0.001) more likely to close than for-profit homes with the same rating (see online supplemental table A8).

**Figure 3:**
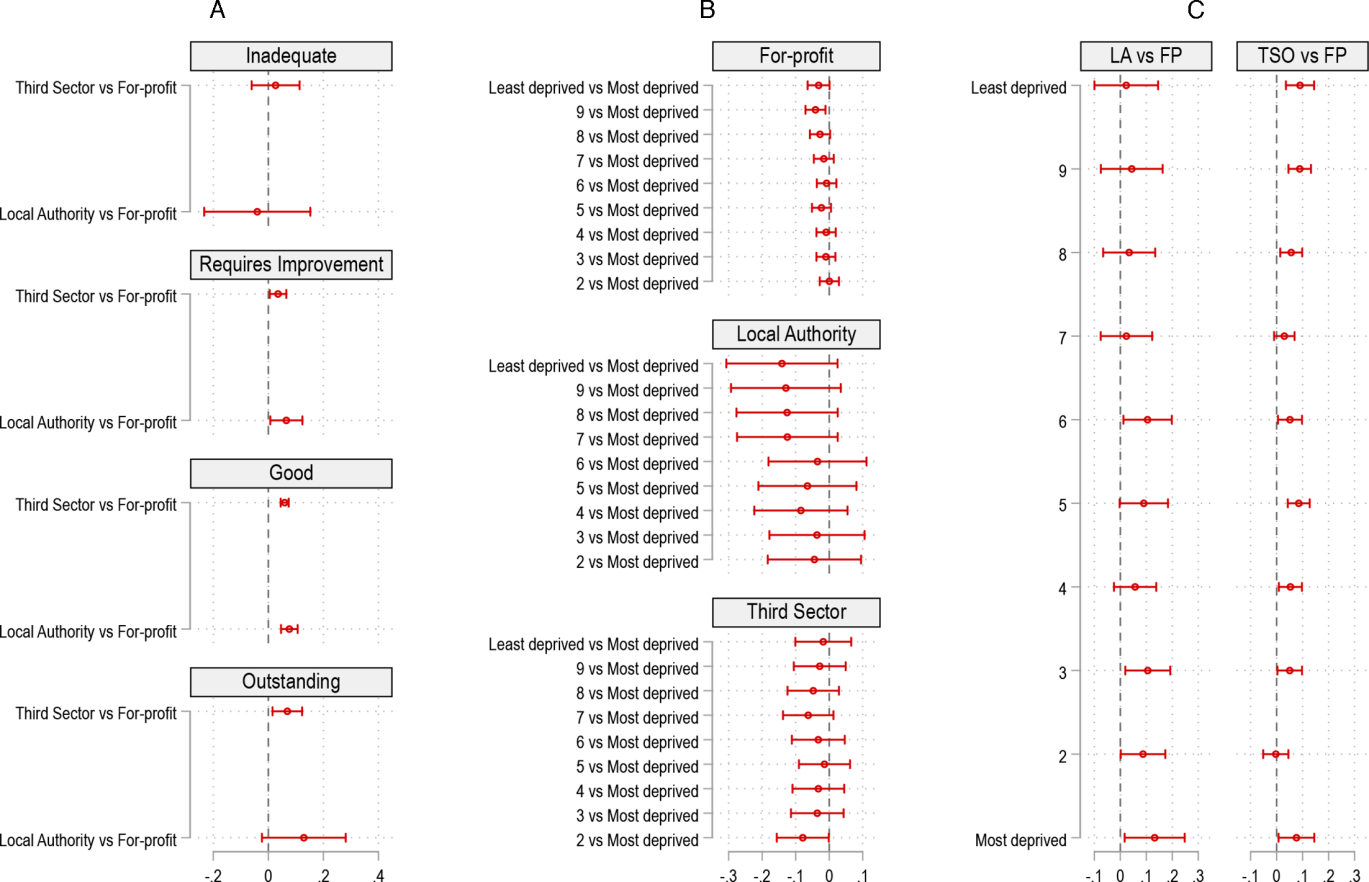
Differences in predicted probabilities of closure across inspection ratings, ownership and area deprivation. This figure models the interaction effect of closure for ownership and quality, and ownership and area deprivation. Panel (A) shows the difference in closure likelihood between third/public sector versus for-profit (FP) homes across different inspection ratings. (B) The differences in closure likelihood between deprivation deciles. (C) The difference in closure likelihood between third/public sector versus for-profit homes across different deprivation deciles. The model controls for the full range of covariates and fixed effects as the adjusted model in table 2. LA, local authority; TSO, third sector organisation.

Additionally, the risk of closure was, on average, higher in the most deprived areas for for-profit homes, but most of the differences were not statistically significant (online supplemental table A5). However, the closure probability across deprivation decile varied by ownership: LA and third sector homes were generally more likely to close than for-profit homes in the more deprived places in England (figure 3). For example, public and third sector homes were 13.2%-points (p=0.025) and 7.6%-points (p=0.030), respectively, more likely to close than for-profit homes in the most deprived decile (online supplemental table A9). In the least deprived decile, this difference was not statistically significant between public and for-profit provision, whereas third sector homes are 9.0%-points (p=0.001) more likely to close than for-profit homes. The differences in the risk of closure were not statistically significant by deprivation deciles between third and public sector homes.

### Inspection ratings of active and closed homes

**Figure 4:**
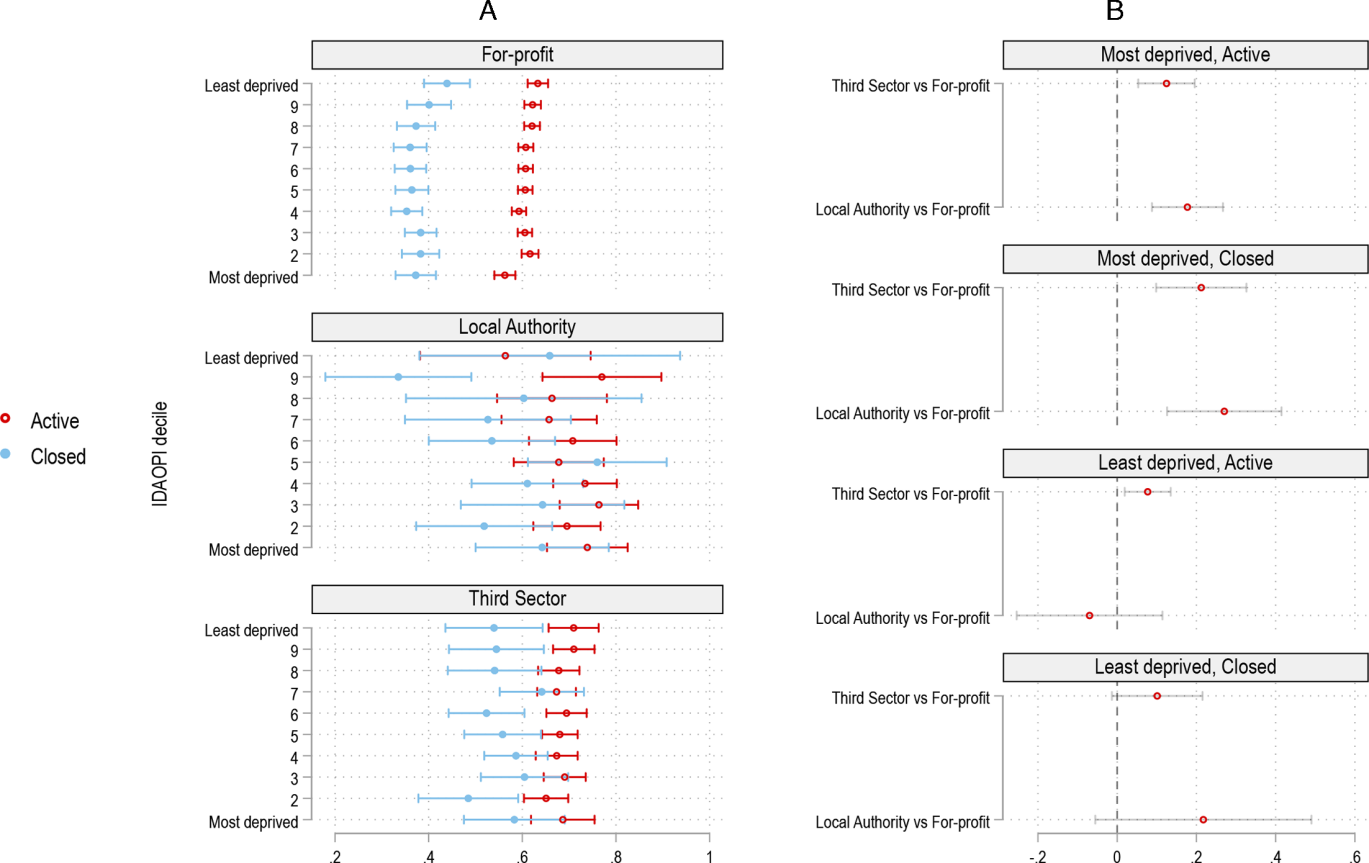
Predicted probability of being rated ‘outstanding’/’good’ versus ‘requires improvement’/’inadequate’ across closure status (active/closed), ownership and area deprivation. This figures visualises the three-way interaction results between ownership, closure status (closed/active) and area deprivation. The outcome for all models is inspection rating (‘inadequate/’requires improvement’ vs ‘good’/’outstanding’). Panel (A) shows the predicted probabilities for being rated ‘good’ or ‘outstanding’ by ownership and area deprivation. Panel (B) shows the difference in predicted probabilities between public/third sector and for-profit provision for closed and active homes and for the least and most deprived deciles. The model controls for the full range of covariates and fixed effects as the adjusted model in table 2. See online supplemental figure A5 for the raw distribution and online supplemental tables A6 and A11 for the difference in predicted probabilities. IDAOPI, Income Deprivation Affecting Older People Index.

The difference in inspection ratings between closed and active providers was substantially larger for for-profit homes, whereas the quality of closed/active public and third sector homes did not generally vary statistically significantly from one another (figure 4A). Care homes ratings were, on average, worst in the most deprived areas, but in our models we find that this is only the case for active for-profit homes. Active for-profit homes in the most deprived decile are more likely to be rated poorly compared with the least deprived decile.

Quality differences between for-profit and third and public sector homes were highest in the most deprived areas for both active and closed homes (figure 4B). Active public and third sector homes in the most deprived decile were 17.7%-points (p<0.001) and 12.4%-points (p<0.001) more likely to be rated ‘outstanding’ or ‘good’ compared with ‘requires improvement’ or ‘inadequate’ than for-profit provision (online supplemental table A11). In the least deprived decile, these differences are substantially reduced, with active third sector homes being 7.7%-points (p=0.002) more likely to be rated ‘good/outstanding’ than for-profit provision. These differences were even larger in closed homes, suggesting that good homes have closed in deprived areas. The difference between active public and for-profit provision in the least deprived decile is not statistically significant.

## Discussion

This is the first longitudinal study of care home closures in England that uses complete data on all care home closures, 2011–2023, and inspection ratings, 2014–2023. Our results can be summarised by five findings. First, since 2011, the closure rate of for-profit, public and third sector care homes has remained fairly consistent, but because fewer third and public sector homes have opened relative to for-profit homes, public and third sector provision has substantially diminished over time. Second, our results show that care homes were more likely to close if they are (a) operated by the public or third sector, (b) located in a deprived area or (c) rated poorly by regulator inspections. Third, we found that public and third sector homes rated the same as for-profit homes are generally more likely to close. Fourth, the closure of public and third sector homes appears to have negatively impacted the quality of care in the most deprived areas. We find that care home inspection ratings are, on average, worst in the most deprived areas, but only for active for-profit care homes. Fifth, the best active homes in deprived areas are operated by public and third sector homes. We do not find evidence that inspection ratings for public and third sector homes are influenced by area deprivation.

This may have implications for aggregate provision. Reports by both the CQC, interest groups, and other regulators have found that adult social care is struggling to meet demand, and the sector has continuously been diagnosed as fragile, neglected and underfunded.^33^,^35^ This development is often reported to be due in part to inadequate public funding and neglect, which has created undesirable knock-on effects on the two-tier system of state-funded and self-funded residents. It has long been argued by the sector that funding for state-funded residents is insufficient,^34 36^ and research has shown that care homes are increasingly forced to cater to self-funded residents for financial survival.^37^ If the survival of care homes is determined by access to self-funders, this will exacerbate inequality in market-based provision. At worst, it can lead to severe equity issues, in which competition for quality primarily applies to prosperous areas, leaving residents in poorer areas without access to the care they need. Data from the Office of National Statistics confirms that care homes in less deprived areas have a higher proportion of self-funders.^38^ Our results corroborate this development in two ways.

First, we found that area deprivation is a significant determinant of closure, and care homes of all ownership types are more likely to close if they operate in deprived areas. This underscores an enduring association between area deprivation and care home survival, even after adjusting for quality and ownership. Second, despite the higher average quality of care provided by public and third sector homes in deprived areas compared with for-profit providers, these homes are at a higher risk of closure. This raises concerns about the vulnerability of care homes that provide high-quality and much-needed care but operate in economically disadvantaged regions. The implications are unsettling—the performance of public and third sector care homes seemingly does not shield them from closure, highlighting the challenges reported by the CQC in sustaining access to high-quality care in deprived areas.^24^ Addressing this issue is critical to avoid exacerbating existing inequalities and ensuring that residents have continued access to the care they need.

## Limitations

The main limitation of this work is the lack of existing data on potential key variables at care home and resident level. These can be summarised by the following points. First, our analysis does not include information on the funding status of residents nor on care home occupancy rates or top-up fees, as such statistics are not currently available at provider level over time. Most care homes serve a mix of state-funded and self-funded residents,^8^ and the lack of analysis on how this influences care provision is a key gap in the literature. Second, financial information on care homes is not publicly available, and our analyses, therefore, do not include variables related to the profit, debt and deficits of care homes, which may influence performance. Third, it is well known that the sector struggles to retain qualified staff and that there is widespread staff shortages.^39^ There is some evidence suggesting that work conditions are poorer in the private sector,^40^ but more work is needed to explore this association. Fourth, the impact of a care home closure can be severe and traumatic for residents.^16^ While our work has revealed important insights in terms of the ownership-specific patterns in the determinants of closures, more work is needed to document the consequences associated with care home closures on residents and LAs.^5^ Most closures involve personal, economic and health costs, which are, therefore, key outcomes in the evaluation of the marketisation of care.

## Conclusion

Our findings suggest that while quality and deprivation are key determinants of care home closures, they operate differently for different types of care homes, which provide new insights into the functioning of the adult social care sector in England and its use of outsourcing. Outsourcing was implemented with the intention to enable the best provider to operate, irrespective of ownership.^13^ Existing work on the determinants of care home closures has found that quality matters for the survival of care homes,^22 23^ which at face value appears in line with the intention of competition. However, past research has also shown that quality of care is typically lower among for-profit homes.^3 11^ At the same time, for-profit provision has continued to grow. To explain this contradiction, we find that the determinants of closure vary according to ownership, which suggests that ownership does matter and that the policy intention to dissolve the distinction between for-profit, public and third sector ownership^13^ has not succeeded. The difference in quality between active and closed public/third sector homes was substantially smaller compared with for-profit homes, suggesting that public and third sector provisions have closed despite their ratings. Furthermore, it appears that churn in the for-profit sector has not resulted in better performance, as active for-profit homes continue to underperform other ownership types, especially in the most deprived areas.

## supplementary material

10.1136/bmjph-2024-001227 online supplemental file 1

## Data Availability

Data are available in a public, open access repository. All data relevant to the study are included in the article or uploaded as online supplemental information.
