# Supplementary material for: Does outsourcing enable the survival of good care homes? A longitudinal analysis of all care homes in England, 2011–2023
Source: BMJ Public Health. 2024 Jul 29;2(2):e001227. doi: 10.1136/bmjph-2024-001227 (PMC11816439; doi:10.1136/bmjph-2024-001227)

Appendix

Figure A1:

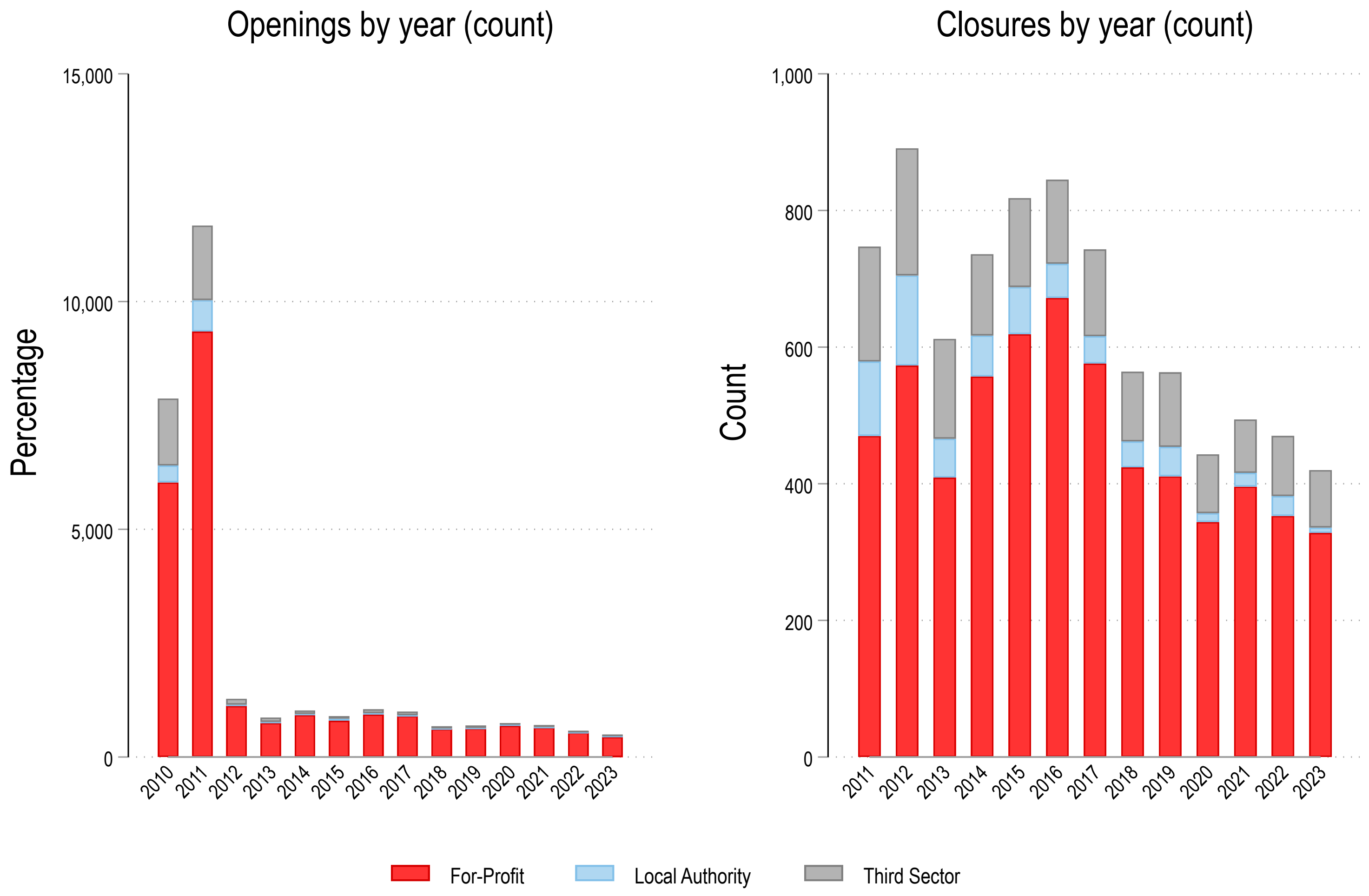

Figure A2: Development in the number and percentage of active for-profit, public, and third sector care homes and registered beds over time.

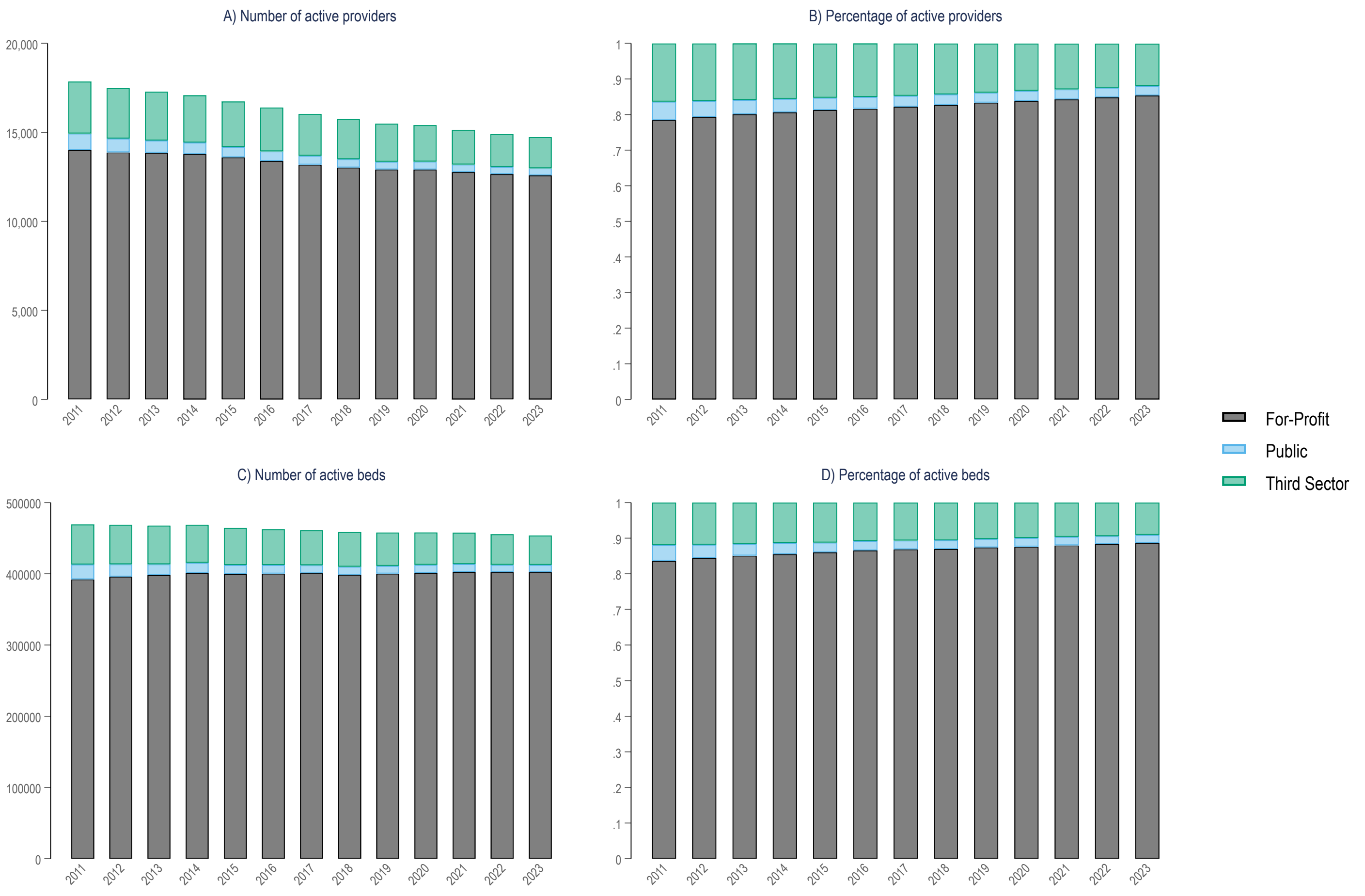

Figure A3: Visualisation of the determinants of care home closure at inspection and local level.

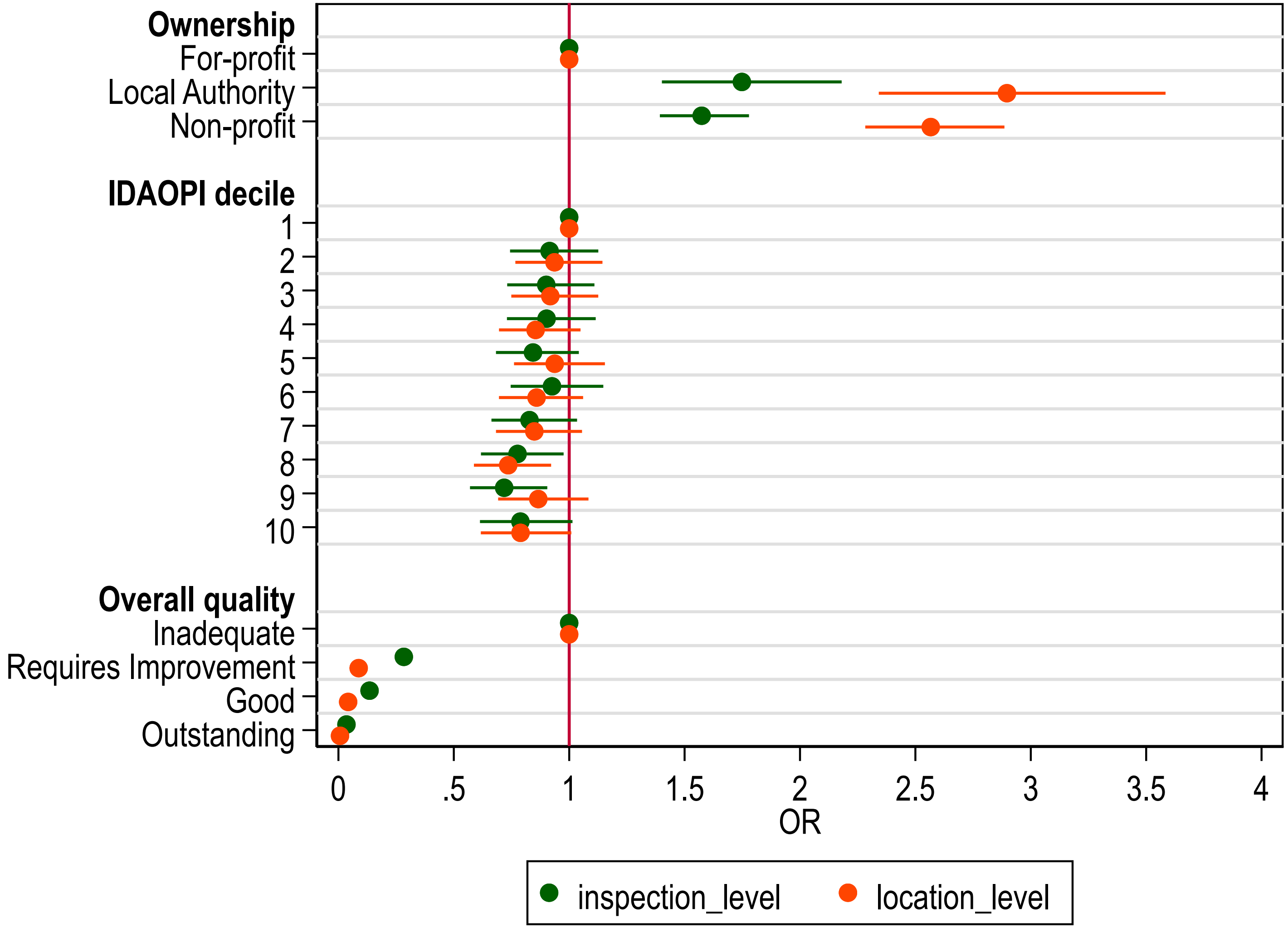

Figure A4: Care home closure determinants by alternative inspection domains.

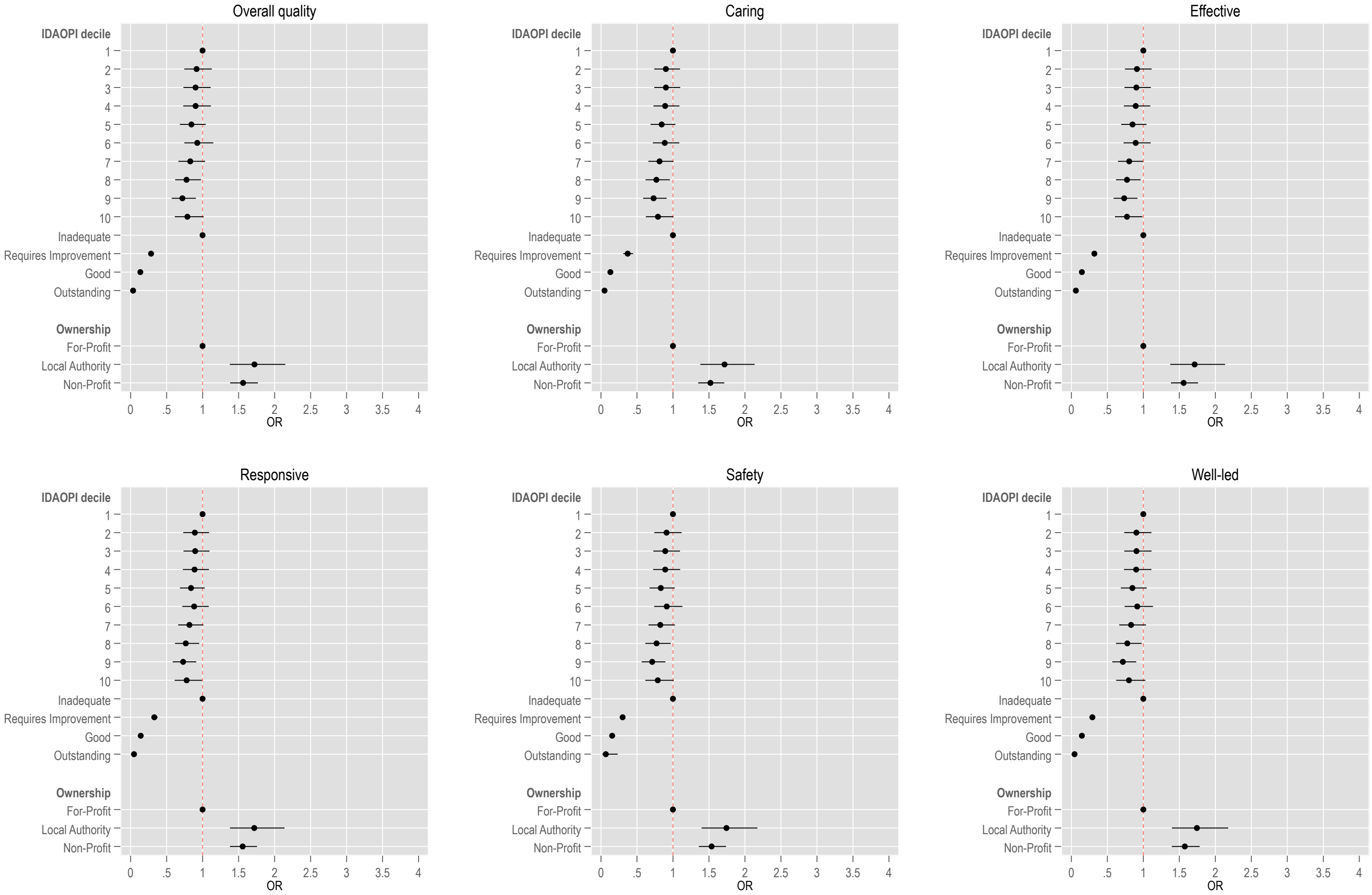

Table A1: Full logistic regression results on the determinants of care home closure.

	(1)	(2)	(3)	(4)
Ownership (reference: for-profit)				
Local Authority	1.427***			1.796***
	[1.156,1.762]			[1.439,2.242]
Third sector	1.374***			1.553***
	[1.227,1.538]			[1.373,1.756]
IDAOPI (%)		1.007**		1.008**
		[1.002,1.011]		[1.003,1.013]
Overall quality (reference: inadequate)				
Requires Improvement			0.305***	0.281***
			[0.280,0.332]	[0.258,0.306]
Good			0.183***	0.133***
			[0.166,0.201]	[0.120,0.146]
Outstanding			0.0421***	0.0345***
			[0.0274,0.0647]	[0.0225,0.0530]
Registered Nursing Services				1.133
				[0.997,1.288]
Registered Dementia Services				0.972
				[0.863,1.094]
Individual/partnership				1.665***
				[1.472,1.884]
Registered mental health service				0.875*
				[0.788,0.972]
Registered disability services				0.865**
				[0.781,0.957]
Number of Registered Beds				0.979***
				[0.975,0.983]
Care homes for older people				0.947
				[0.839,1.070]
Months of registration				0.999
				[0.998,1.001]
Observations	65726	65726	58980	58967
Pseudo R^2	.057	.055	.086	.128
Care home clusters	18,379	18,379	18,323	18,309
LA fixed effects	Yes	Yes	Yes	Yes
Inspection year fixed effects	Yes	Yes	Yes	Yes

*95% confidence intervals in parentheses. *** p<.001, ** p<.01, * p<.05.* All standard errors are clustered at provider and care home level.
Note that the months of registration take provider takeovers into account and are based on the difference between the start and inspection date.

Table A2: Logistic regression (at care home level) examining care home and resident characteristics associated with care home closures.

	Care home level			
	(1)	(2)	(3)	(4)
Ownership (reference: for-profit)				
Local Authority	3.112***			2.83***
	(.184)			(.307)
Third sector	2.015***			2.559***
	(.076)			(.152)
IDAOPI (%)		1.009***		1.013***
		(.001)		(.002)
Latest overall rating (reference: inadequate)				
Requires Improvement			.098***	.09***
			(.008)	(.008)
Good			.049***	.044***
			(.004)	(.004)
Outstanding			.006***	.007***
			(.002)	(.002)
Registered Nursing Services				1.105
				(.066)
Registered Dementia Services				.801***
				(.046)
Individual or partnership				2.238***
				(.135)
Number of Registered Beds				.983***
				(.002)
Active locations				.997***
				(.001)
Registered mental health service				.802***
				(.04)
Registered disability services				.667***
				(.032)
Care homes for older people				.843**
				(.047)
Months of registration				.985***
				(0)
Observations	29468	29468	21511	21509
Pseudo R^2	.033	.016	.12	.226
Care home clusters	23,005	23,005	18,337	18,335
LA fixed effects	Yes	Yes	Yes	Yes

*Robust standard errors are in parentheses. ***p<.001, **p<.01, *p<.05.* All standard errors are clustered at provider and care home level. Note that the months of registration take provider takeovers into account and are based on the difference between the start and end date in the location models. We used the IDAOPI 2015 score for locations that opened from 2011-2017 and the IDAOPI 2019 score for those opening in 2018-2023.

Table A3: Interaction results of ownership and overall quality and area deprivation and quality on closure – models 2 and 4 are used to calculate the marginal effects in Figures 2 and 3 in the manuscript.

	(1)	(2)	(3)	(4)
**Interaction: ownership and quality (continuous)**	1.046**			
	(.015)			
**Interaction: ownership & overall quality (categorical)**				
(Reference: For-Profit#inadequate)				
For-Profit#Requires Improvement		.283***		
		(.013)		
For-Profit#Good		.126***		
		(.007)		
For-Profit#Outstanding		.021***		
		(.007)		
Local Authority#Inadequate		.822		
		(.395)		
Local Authority#Requires Improvement		.427***		
		(.076)		
Local Authority#Good		.251***		
		(.031)		
Local Authority#Outstanding		.196*		
		(.129)		
Non-Profit#Inadequate		1.135		
		(.239)		
Non-Profit#Requires Improvement		.357***		
		(.037)		
Non-Profit#Good		.22***		
		(.017)		
Non-Profit#Outstanding		.106***		
		(.037)		
Area deprivation IDAOPI (%)		1.008**		
		(.002)		
Overall quality (reference: inadequate)				
Requires Improvement			.284***	.283***
			(.012)	(.012)
Good			.135***	.135***
			(.007)	(.007)
Outstanding			.035***	.035***
			(.008)	(.008)
**Interaction: IDAOPI % * ownership**			1.009***	
			(.001)	
**Interaction: IDAOPI decile * ownership**				1.032***
				(.005)
IDAOPI %	1.009***			
	(.002)			
Registered Nursing Services	1.18**	1.132	1.112	1.114
	(.075)	(.074)	(.072)	(.072)
Registered Dementia Services	1.043	.969	.965	.975
	(.063)	(.059)	(.058)	(.059)
Individual or partnership	1.727***	1.666***	1.609***	1.62***
	(.106)	(.105)	(.1)	(.101)
Number of Registered Beds	.865**	.874*	.874*	.88*
	(.046)	(.047)	(.046)	(.047)
Registered mental health service	.88*	.864**	.875**	.874**
	(.045)	(.045)	(.045)	(.045)
Registered disability services	.982***	.979***	.979***	.979***
	(.002)	(.002)	(.002)	(.002)
Care homes for older people	1.003	.947	.932	.934

	(.062)	(.059)	(.058)	(.058)
Months of registration	1	.999	1	1
	(.001)	(.001)	(.001)	(.001)
Observations	58967	58967	58967	58967
Pseudo R2	.075	.129	.126	.126
Robust standard errors clustered by care home and provider	Yes	Yes	Yes	Yes
LA fixed effects	Yes	Yes	Yes	Yes
Inspection year fixed effects	YEs	Yes	Yes	Yes

Robust standard errors are in parentheses
****p<.001, **p<.01, *p<.05*

Table A4: Three-way interaction logistic regression results between ownership, closure status (closed/active), and area deprivation on care quality ('Inadequate/'Requires improvement' versus 'Good'/'Outstanding') - used to calculate the marginal effects in Figure 4.

	**'Inadequate/Requires improvement' (reference) versus 'Good/Outstanding'**
For-profit # Most deprived # Active	1
	[1,1]
For-profit # Most deprived # Closed	0.431***
	[0.347,0.535]
For-profit # 2 # Active	1.273***
	[1.126,1.439]
For-profit # 2 # Closed	0.452***
	[0.368,0.555]
For-profit # 3 # Active	1.211**
	[1.075,1.365]
For-profit # 3 # Closed	0.453***
	[0.376,0.544]
For-profit # 4 # Active	1.144*
	[1.014,1.290]
For-profit # 4 # Closed	0.394***
	[0.327,0.475]
For-profit # 5 # Active	1.215**
	[1.075,1.374]
For-profit # 5 # Closed	0.415***
	[0.342,0.503]
For-profit # 6 # Active	1.220**
	[1.079,1.380]
For-profit # 6 # Closed	0.409***
	[0.339,0.493]
For-profit # 7 # Active	1.222**
	[1.079,1.385]
For-profit # 7 # Closed	0.408***
	[0.335,0.495]
For-profit # 8 # Active	1.299***
	[1.142,1.478]
For-profit # 8 # Closed	0.432***
	[0.349,0.537]
For-profit # 9 # Active	1.306***
	[1.145,1.490]
For-profit # 9 # Closed	0.491***
	[0.387,0.622]
For-profit # Least deprived # Active	1.375***
	[1.190,1.590]
For-profit # Least deprived # Closed	0.582***
	[0.458,0.741]
Local Authority # Most deprived # Active	2.342***

	[1.440,3.809]
Local Authority # Most deprived # Closed	1.436
	[0.731,2.822]
Local Authority # 2 # Active	1.860**
	[1.276,2.712]
Local Authority # 2 # Closed	0.824
	[0.435,1.563]
Local Authority # 3 # Active	2.699***
	[1.628,4.475]
Local Authority # 3 # Closed	1.442
	[0.629,3.304]
Local Authority # 4 # Active	2.279***
	[1.549,3.353]
Local Authority # 4 # Closed	1.241
	[0.714,2.156]
Local Authority # 5 # Active	1.704*
	[1.048,2.770]
Local Authority # 5 # Closed	2.644*
	[1.104,6.334]
Local Authority # 6 # Active	1.982**
	[1.207,3.255]
Local Authority # 6 # Closed	0.886
	[0.487,1.612]
Local Authority # 7 # Active	1.541
	[0.935,2.538]
Local Authority # 7 # Closed	0.853
	[0.391,1.862]
Local Authority # 8 # Active	1.587
	[0.892,2.825]
Local Authority # 8 # Closed	1.198
	[0.381,3.762]
Local Authority # 9 # Active	2.804**
	[1.297,6.063]
Local Authority # 9 # Closed	0.361**
	[0.169,0.775]
Local Authority # Least deprived # Active	1.004
	[0.446,2.264]
Local Authority # Least deprived # Closed	1.551
	[0.405,5.936]
Third Sector # Most deprived # Active	1.779**
	[1.253,2.525]
Third Sector # Most deprived # Closed	1.094
	[0.671,1.782]
Third Sector # 2 # Active	1.494**
	[1.172,1.905]
Third Sector # 2 # Closed	0.711
	[0.442,1.143]
Third Sector # 3 # Active	1.815***
	[1.419,2.321]
Third Sector # 3 # Closed	1.206
	[0.782,1.861]
Third Sector # 4 # Active	1.667***
	[1.312,2.119]
Third Sector # 4 # Closed	1.111
	[0.808,1.529]
Third Sector # 5 # Active	1.722***
	[1.392,2.132]
Third Sector # 5 # Closed	0.979
	[0.673,1.424]
Third Sector # 6 # Active	1.851***
	[1.456,2.353]
Third Sector # 6 # Closed	0.842

	[0.583,1.217]
Third Sector # 7 # Active	1.665***
	[1.327,2.088]
Third Sector # 7 # Closed	1.431
	[0.926,2.212]
Third Sector # 8 # Active	1.704***
	[1.339,2.168]
Third Sector # 8 # Closed	0.908
	[0.579,1.425]
Third Sector # 9 # Active	2.005***
	[1.556,2.584]
Third Sector # 9 # Closed	0.924
	[0.585,1.458]
Third Sector # Least deprived # Active	2.002***
	[1.487,2.694]
Third Sector # Least deprived # Closed	0.903
	[0.566,1.440]
Registered Nursing Services	0.836***
	[0.792,0.882]
Registered Dementia Services	0.772***
	[0.730,0.816]
Individual or partnership	0.825***
	[0.770,0.884]
Registered mental health service	0.977
	[0.929,1.027]
Registered disability services	1.024
	[0.978,1.073]
Number of Registered Beds	0.992***
	[0.990,0.993]
Care homes for older people	0.700***
	[0.656,0.748]
Months of registration	0.999***
	[0.998,0.999]
Constant	5.650***
	[2.762,11.56]
Observations	58973

The outcome for all models is inspection rating ('Inadequate/'Requires improvement' versus 'Good'/'Outstanding'). The model controls for the full range of covariates and fixed effects as the adjusted model in Table 2.
Exponentiated coefficients; 95% confidence intervals in brackets
* $p < 0.05$, ** $p < 0.01$, *** $p < 0.001$

Table A5: Changes in the predicted closure probabilities and p values by ownership and quality.

	**Public sector**		**Third sector**		**For-profit**	
**Quality**	Change	p-value	Change	p-value	Change	p-value
Requires Improvement vs Inadequate	-0.125	0.194	-0.222	0	-0.231	0
Good vs Inadequate	-0.207	0.033	-0.291	0	-0.324	0
Outstanding vs Inadequate	-0.239	0.056	-0.365	0	-0.408	0
Good vs Requires Improvement	-0.082	0.003	-0.069	0	-0.093	0
Outstanding vs Requires Improvement	-0.114	0.169	-0.143	0	-0.177	0
Outstanding vs Good	-0.031	0.688	-0.074	0.006	-0.084	0

Table A6: Changes in the predicted closure probabilities and p values by ownership and IDAOPI decile.

	**Public sector**		**Third sector**		**For-profit**	
	Change	p-value	Change	p-value	Change	p-value
**IDAOPI deciles**						
1 vs 2	-0.044	0.533	-0.079	0.046	0	0.975
1 vs 3	-0.036	0.615	-0.036	0.374	-0.01	0.492
1 vs 4	-0.084	0.234	-0.032	0.41	-0.009	0.53
1 vs 5	-0.065	0.384	-0.014	0.718	-0.023	0.111
1 vs 6	-0.035	0.64	-0.033	0.42	-0.008	0.604
1 vs 7	-0.125	0.103	-0.063	0.103	-0.016	0.288
1 vs 8	-0.126	0.103	-0.048	0.225	-0.028	0.073
1 vs 9	-0.129	0.122	-0.028	0.474	-0.041	0.007
1 vs 10	-0.141	0.096	-0.018	0.676	-0.032	0.058

Table A7: Changes in the predicted probabilities to receive different levels of inspection ratings by ownership and IDAOPI.

	Inadequate	Requires improvement	Good	Outstanding
**IDAOPI**				
		**For-profit**		
2 vs 1	-0.015	-0.031	0.043	0.003
p-value	0	0	0	0
3 vs 1	-0.012	-0.024	0.033	0.002
p-value	0.005	0.004	0.004	0.003
4 vs 1	-0.008	-0.016	0.023	0.001
p-value	0.051	0.046	0.048	0.044
5 vs 1	-0.013	-0.025	0.036	0.002
p-value	0.004	0.003	0.003	0.002
6 vs 1	-0.011	-0.021	0.03	0.002
p-value	0.014	0.012	0.012	0.011
7 vs 1	-0.012	-0.025	0.035	0.002
p-value	0.005	0.004	0.005	0.004
8 vs 1	-0.015	-0.032	0.044	0.003
p-value	0.001	0	0	0
9 vs 1	-0.019	-0.041	0.057	0.004
p-value	0	0	0	0
10 vs 1	-0.023	-0.05	0.068	0.005
p-value	0	0	0	0
		**Public**		
2 vs 1	0.01	0.042	-0.043	-0.009
p-value	0.19	0.191	0.188	0.212
3 vs 1	-0.002	-0.01	0.009	0.003
p-value	0.775	0.775	0.775	0.776
4 vs 1	0.006	0.027	-0.027	-0.006
p-value	0.404	0.406	0.403	0.418
5 vs 1	0.002	0.007	-0.007	-0.002
p-value	0.872	0.871	0.871	0.87
6 vs 1	0.01	0.041	-0.041	-0.009
p-value	0.257	0.251	0.253	0.26
7 vs 1	0.019	0.076	-0.081	-0.015
p-value	0.056	0.044	0.047	0.052
8 vs 1	0.013	0.053	-0.055	-0.011
p-value	0.255	0.23	0.242	0.211
9 vs 1	0.017	0.068	-0.071	-0.013
p-value	0.208	0.168	0.186	0.138
10 vs 1	0.024	0.092	-0.099	-0.017
p-value	0.222	0.151	0.182	0.09
		**Third sector**		
2 vs 1	0.011	0.041	-0.045	-0.007
p-value	0.087	0.092	0.088	0.113
3 vs 1	-0.002	-0.007	0.008	0.002
p-value	0.759	0.758	0.758	0.755
4 vs 1	0	0	0	0
p-value	0.984	0.984	0.984	0.984
5 vs 1	0.002	0.009	-0.01	-0.002
p-value	0.695	0.697	0.696	0.702
6 vs 1	0.003	0.013	-0.013	-0.002
p-value	0.592	0.595	0.593	0.603
7 vs 1	0.001	0.003	-0.003	0
p-value	0.916	0.916	0.916	0.916
8 vs 1	0.004	0.014	-0.015	-0.003
p-value	0.551	0.555	0.553	0.563
9 vs 1	-0.001	-0.004	0.004	0.001
p-value	0.859	0.859	0.859	0.858
10 vs 1	-0.002	-0.009	0.009	0.002
p-value	0.742	0.741	0.742	0.74

Table A8: Difference in predicted closure probabilities across ownership for different inspection ratings.

	Change	p-value
**Inadequate**		
Local Authority vs For-Profit	-0.04	0.683
Non-Profit vs For-Profit	0.027	0.551
Non-Profit vs Local Authority	0.067	0.532
**Requires improvement**		
Local Authority vs For-Profit	0.065	0.028
Non-Profit vs For-Profit	0.035	0.021
Non-Profit vs Local Authority	-0.03	0.353
**Good**		
Local Authority vs For-Profit	0.076	0
Non-Profit vs For-Profit	0.059	0
Non-Profit vs Local Authority	-0.017	0.303
**Outstanding**		
Local Authority vs For-Profit	0.129	0.097
Non-Profit vs For-Profit	0.069	0.012
Non-Profit vs Local Authority	-0.06	0.464

Table A9: Difference in predicted closure probabilities across ownership for different IDAOPI deciles

	Difference	P-value
(Local Authority vs For-profit) IDAOPI 1	0.1322502	0.025
(Local Authority vs For-profit) IDAOPI 2	0.0875405	0.045
(Local Authority vs For-profit) IDAOPI 3	0.1057049	0.017
(Local Authority vs For-profit) IDAOPI 4	0.0570249	0.169
(Local Authority vs For-profit) IDAOPI 5	0.0903761	0.057
(Local Authority vs For-profit) IDAOPI 6	0.1050504	0.027
(Local Authority vs For-profit) IDAOPI 7	0.0235795	0.641
(Local Authority vs For-profit) IDAOPI 8	0.0341689	0.504
(Local Authority vs For-profit) IDAOPI 9	0.0441231	0.467
(Local Authority vs For-profit) IDAOPI 10	0.0229353	0.714
(Third Sector vs For-profit) IDAOPI 1	0.0762264	0.03
(Third Sector vs For-profit) IDAOPI 2	-0.0030286	0.903
(Third Sector vs For-profit) IDAOPI 3	0.0504975	0.036
(Third Sector vs For-profit) IDAOPI 4	0.0530763	0.019
(Third Sector vs For-profit) IDAOPI 5	0.085249	0
(Third Sector vs For-profit) IDAOPI 6	0.0514753	0.029
(Third Sector vs For-profit) IDAOPI 7	0.0297649	0.139
(Third Sector vs For-profit) IDAOPI 8	0.0562238	0.009
(Third Sector vs For-profit) IDAOPI 9	0.0889839	0
(Third Sector vs For-profit) IDAOPI 10	0.0901095	0.001

Table A10: Difference in predicted closure probabilities to be rated good/outstanding across ownership and for active/closed homes

	Difference	P-value
**Closed homes**		
Local Authority vs For-Profit	0.21	0.000
Non-Profit vs For-Profit	0.187	0.000
Non-Profit vs Local Authority	-0.022	0.476
**Active homes**		
Local Authority vs For-Profit	0.094	0.000
Non-Profit vs For-Profit	0.076	0.000
Non-Profit vs Local Authority	-0.017	0.343

Table A11: Difference in predicted closure probabilities to be rated good/outstanding across ownership in the most and least deprived IDAOPI deciles.

	Difference	P-value
**IDAOPI =1, Active homes**		
Local Authority vs For-Profit	0.177	0
Non-Profit vs For-Profit	0.124	0.001
Non-Profit vs Local Authority	-0.053	0.351
**IDAOPI =1, Closed homes**		
Local Authority vs For-Profit	0.27	0
Non-Profit vs For-Profit	0.212	0
Non-Profit vs Local Authority	-0.058	0.511
**IDAOPI =10, Active homes**		
Local Authority vs For-Profit	-0.07	0.457
Non-Profit vs For-Profit	0.077	0.009
Non-Profit vs Local Authority	0.147	0.13
**IDAOPI =10, Closed homes**		
Local Authority vs For-Profit	0.218	0.118
Non-Profit vs For-Profit	0.101	0.083
Non-Profit vs Local Authority	-0.117	0.427

Figure A5: Distribution of inspection outcomes across ownership and deprivation deciles.

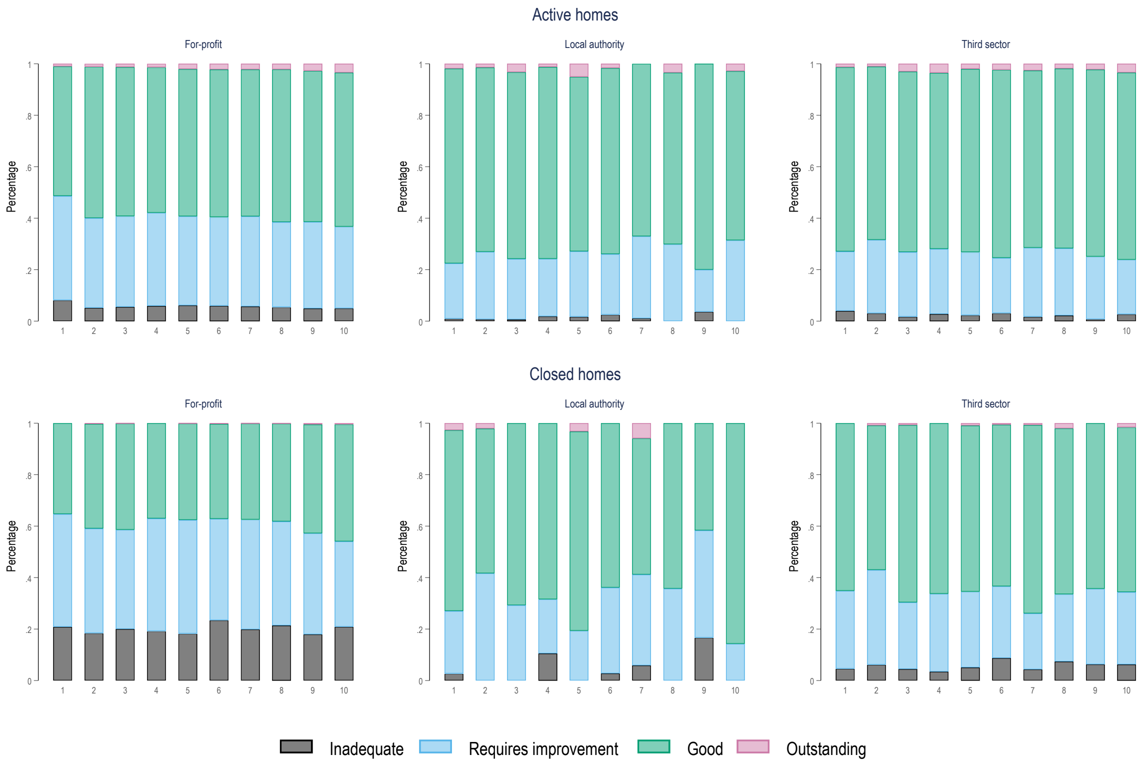

Figure A6: Alternative model specification of Figure 4 in the manuscript, using ordered logistic regression and all inspection rating domains.

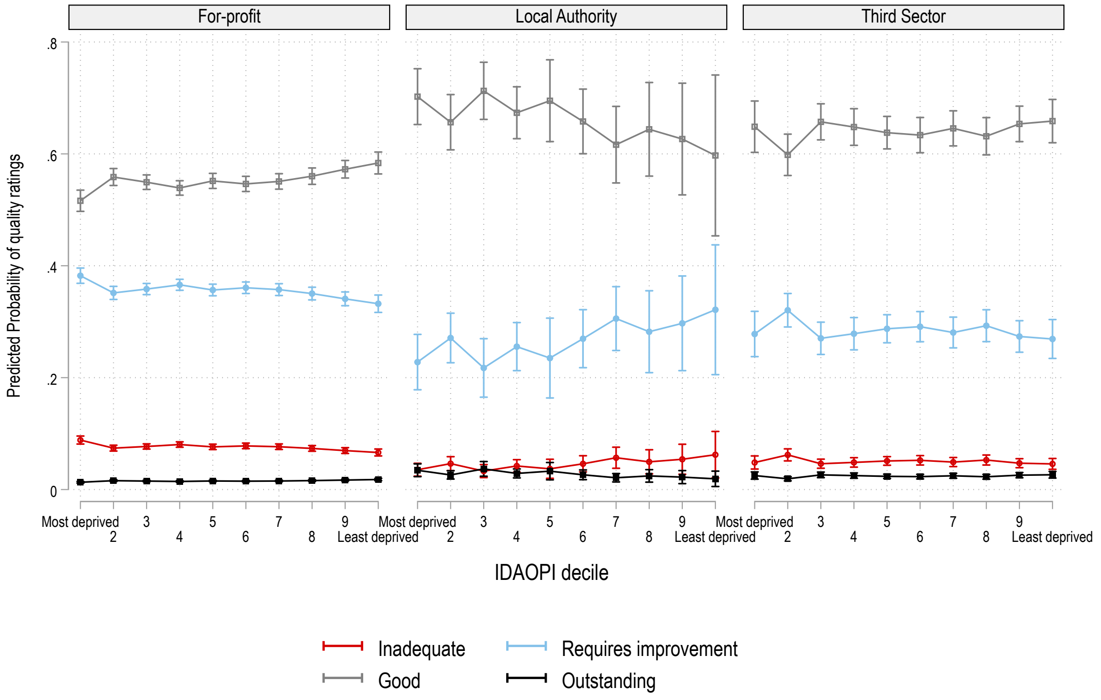

Figure A7: Alternative model specification of Figure 4 in the manuscript, using ordered logistic regression and all inspection rating domains – separated by ownership and closure status

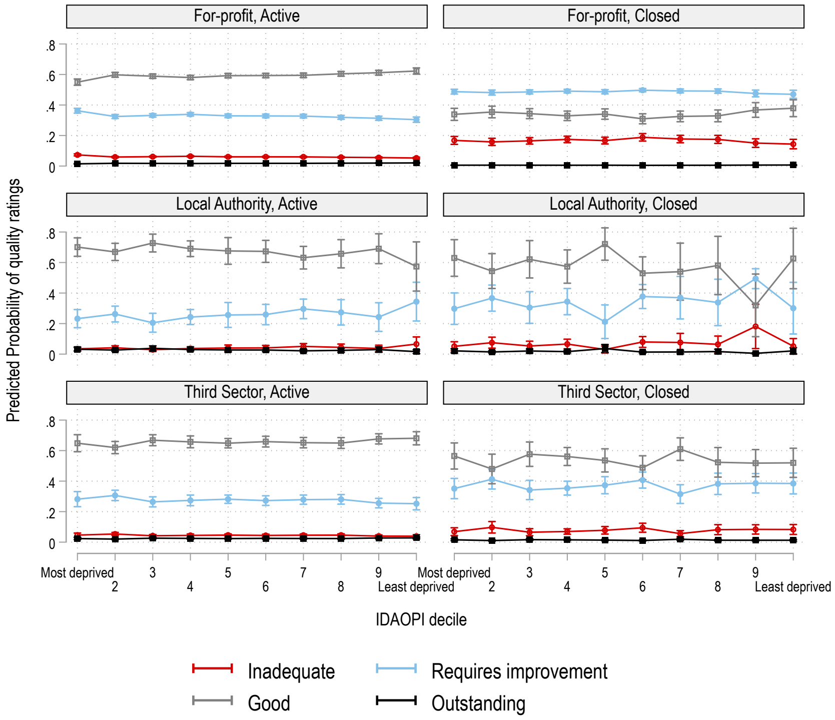

Supplement: online supplemental file 1 [file bmjph-2-2-s001.pdf]
